# Supplementary material for: Centralized Modularity of N-Linked Glycosylation Pathways in Mammalian Cells
Source: PLoS One. 2009 Oct 5;4(10):e7317. doi: 10.1371/journal.pone.0007317 (PMC2750756; doi:10.1371/journal.pone.0007317)
Supplement: Table S1 — Lists of enhanced or suppressed modules under combinations of enzyme regulation. For the comparison with Figure 3(B), the most right column labels each regulatory outcome in which no more than six modules become enhanced. (0.09 MB PDF) [file pone.0007317.s001.pdf]

Regulated Enzyme		Affected Module		
Up-regulated	Down-regulated	Enhanced	Suppressed	
	GnTII, GnTIII, FucT	1	2~21	1
	GnTII, GnTIII, GnTIV, FucT	1	2~21	
	GnTII, GnTIII, GnTIV, GnTV, FucT	1	2~21	
	GnTII, GnTIII, GnTV, FucT	1	2~21	
	GnTIII, GnTIV, GnTV, FucT	1	2~21	
	GnTII, GnTIII, GnTIV	1, 2	3~21	2
	GnTII, GnTIII, GnTIV, GnTV	1, 2	3~21	
	GnTIII, GnTIV, GnTV	1, 2	3~21	
	GnTII, GnTIV, FucT	1, 3	2, 4~21	3
	GnTII, GnTIV, GnTV, FucT	1, 3	2, 4~21	
	GnTIII, GnTIV, FucT	1, 16	2~15, 17~21	4
	GnTIII, GnTV, FucT	1, 17	2~16, 18~21	5
	GnTII, FucT	1, 3, 5	2, 4, 6~21	6
	GnTII, GnTV, FucT	1, 3, 5	2, 4, 6~21	
	GnTIV, GnTV, FucT	1, 3, 7	2, 4~6, 8~21	7
	GnTII, GnTIII	1, 2, 9	3~8, 10~21	8
	GnTII, GnTIII, GnTV	1, 2, 9	3~8, 10~21	
	GnTII, GnTIV	1~4	5~21	9
	GnTII, GnTIV, GnTV	1~4	5~21	
	GnTIII, GnTIV	1, 2, 15, 16	3~14, 17~21	10
	GnTIII, FucT	1, 16~18	2~15, 19~21	11
	GnTIII, GnTV	1, 2, 9, 14, 17	3~8, 10~13, 15, 16, 18~21	12
	GnTIV, FucT	1, 3, 7, 12, 16	2, 4~6, 8~11, 13~15, 17~21	13
	GnTIV, GnTV	1~4, 7, 8	5, 6, 9~21	14
	GnTV, FucT	1, 3, 5, 7, 10, 17	2, 4, 6, 8, 9, 11~16, 18~21	15
	GnTII	1~6, 9	7, 8, 10~21	
	GnTII, GnTV	1~6, 9	7, 8, 10~21	
GnTV		12, 13, 15, 16, 18~21	1~11, 14, 17	
	GnTIII	1, 2, 9, 14~19	3~8, 10~13, 20, 21	
	GnTIV	1~4, 7, 8, 12, 13, 15, 16	5, 6, 9~11, 14, 17~21	
	FucT	1, 3, 5, 7, 10, 12, 16~18, 20	2, 4, 6, 8, 9, 11, 13~15, 19, 21	
FucT		2, 4, 6, 8, 9, 11, 13~15, 19, 21	1, 3, 5, 7, 10, 12, 16~18, 20	
GnTIV		5, 6, 9~11, 14, 17~21	1~4, 7, 8, 12, 13, 15, 16	
GnTIII		3~8, 10~13, 20, 21	1, 2, 9, 14~19	
	GnTV	1~11, 14, 17	12, 13, 15, 16, 18~21	
GnTII		7, 8, 10~21	1~6, 9	
GnTII, GnTV		7, 8, 10~21	1~6, 9	
GnTV, FucT		2, 4, 6, 8, 9, 11~16, 18~21	1, 3, 5, 7, 10, 17	
GnTIV, GnTV		5, 6, 9~21	1~4, 7, 8	
GnTIV, FucT		2, 4~6, 8~11, 13~15, 17~21	1, 3, 7, 12, 16	
GnTIII, GnTV		3~8, 10~13, 15, 16, 18~21	1, 2, 9, 14, 17	
GnTIII, FucT		2~15, 19~21	1, 16~18	
GnTIII, GnTIV		3~14, 17~21	1, 2, 15, 16	
GnTIV, GnTV, FucT		2, 4~6, 8~21	1, 3, 7	
GnTII, FucT		2, 4, 6~21	1, 3, 5	
GnTII, GnTV, FucT		2, 4, 6~21	1, 3, 5	
GnTII, GnTIII		3~8, 10~21	1, 2, 9	
GnTII, GnTIII, GnTV		3~8, 10~21	1, 2, 9	
GalT		1, 2, 5~21	3, 4	

Regulated Enzyme		Affected Module	
Up-regulated	Down-regulated	Enhanced	Suppressed
GnTII, GalT		1, 2, 5~21	3, 4
GnTII, GnTIV, GalT		1, 2, 5~21	3, 4
GnTII, GnTIV, GnTV, GalT		1, 2, 5~21	3, 4
GnTII, GnTV, GalT		1, 2, 5~21	3, 4
GnTIV, GalT		1, 2, 5~21	3, 4
GnTIV, GnTV, GalT		1, 2, 5~21	3, 4
GnTV, GalT		1, 2, 5~21	3, 4
GnTIII, GnTV, FucT		2~16, 18~21	1, 17
GnTIII, GnTIV, FucT		2~15, 17~21	1, 16
GnTII, GnTIV, FucT		2, 4~21	1, 3
GnTII, GnTIV, GnTV, FucT		2, 4~21	1, 3
GnTII, GnTIII, GnTIV		3~21	1, 2
GnTII, GnTIII, GnTIV, GnTV		3~21	1, 2
GnTIII, GnTIV, GnTV		3~21	1, 2
GnTII, GnTIV		5~21	1~4
GnTII, GnTIV, GnTV		5~21	1~4
FucT, GalT		1, 2, 4~21	3
GnTII, FucT, GalT		1, 2, 4~21	3
GnTII, GnTIV, FucT, GalT		1, 2, 4~21	3
GnTII, GnTIV, GnTV, FucT, GalT		1, 2, 4~21	3
GnTII, GnTV, FucT, GalT		1, 2, 4~21	3
GnTIV, FucT, GalT		1, 2, 4~21	3
GnTIV, GnTV, FucT, GalT		1, 2, 4~21	3
GnTV, FucT, GalT		1, 2, 4~21	3
GnTII, GnTIII, FucT		2~21	1
GnTII, GnTIII, GnTIV, FucT		2~21	1
GnTII, GnTIII, GnTIV, GnTV, FucT		2~21	1
GnTII, GnTIII, GnTV, FucT		2~21	1
GnTIII, GnTIV, GnTV, FucT		2~21	1
