# Supplementary figures and images for: Centralized Modularity of N-Linked Glycosylation Pathways in Mammalian Cells
Source: PLoS One. 2009 Oct 5;4(10):e7317. doi: 10.1371/journal.pone.0007317 (PMC2750756; doi:10.1371/journal.pone.0007317)

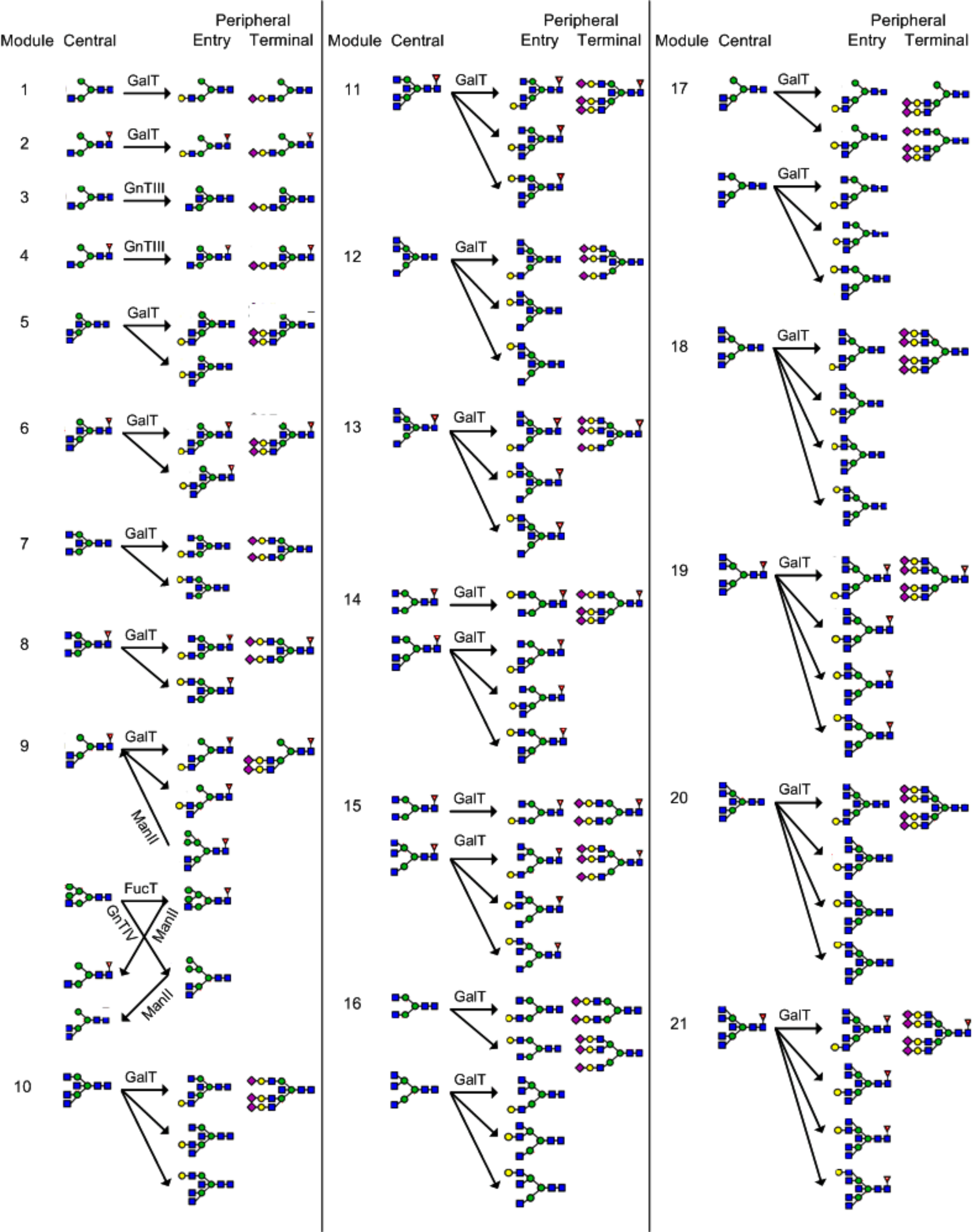

Supplement: Figure S1 — Entry and terminal glycans of peripheral modules. For each module, the parent glycans in the central region and the corresponding reactions are also depicted. The bulk of each module is dominated by galactosylation and sialylation. (0.10 MB PDF) [file pone.0007317.s002.pdf]
