# Supplementary material for: Centralized Modularity of N-Linked Glycosylation Pathways in Mammalian Cells
Source: PLoS One. 2009 Oct 5;4(10):e7317. doi: 10.1371/journal.pone.0007317 (PMC2750756; doi:10.1371/journal.pone.0007317)
Supplement: Figure S2 — Reactions between glycans belonging to different modules. (0.08 MB PDF) [file pone.0007317.s003.pdf]

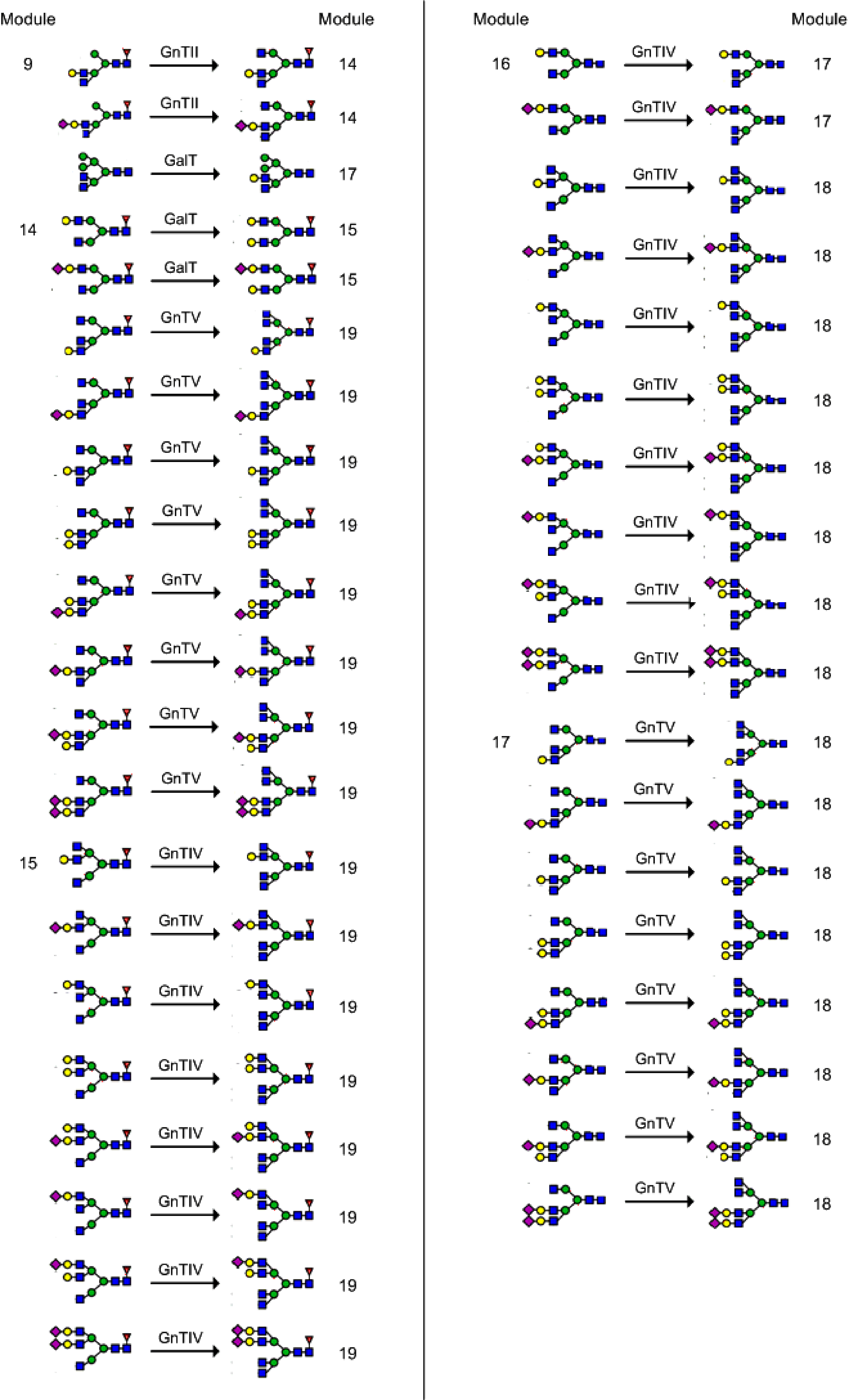
Module
Module
9
GnTII
14
GnTII
14
GalT
17
14
GalT
15
GalT
15
GnTV
19
GnTV
19
GnTV
19
GnTV
19
GnTV
19
GnTV
19
GnTV
19
GnTV
19
15
GnTIV
19
GnTIV
19
GnTIV
19
GnTIV
19
GnTIV
19
GnTIV
19
GnTIV
19
GnTIV
19
Module
Module
16
GnTIV
17
GnTIV
17
GnTIV
18
GnTIV
18
GnTIV
18
GnTIV
18
GnTIV
18
GnTIV
18
GnTIV
18
GnTIV
18
17
GnTV
18
GnTV
18
GnTV
18
GnTV
18
GnTV
18
GnTV
18
GnTV
18
GnTV
18
